# An Assessment of the Longitudinal Construct Validity of the Pain Behavioral Scale (PaBS) in a Saudi Population with Chronic Low Back Pain: A Preliminary Study

**DOI:** 10.3390/healthcare11121743

**Published:** 2023-06-14

**Authors:** Dalia Alimam, Ahmed Alhowimel, Faris Alodaibi, Mazyad Alotaibi, Hosam Alzahrani, Nouf Almutairi, Ali Alqahtani, Lolwah Alrashed Alhumaid, Andrew Leaver, Martin Mackey

**Affiliations:** 1 Health Rehabilitation Sciences, College of Applied Medical Sciences, King Saud University, Riyadh 11451, Saudi Arabia; falodaibi@ksu.edu.sa (F.A.); lalrashed@ksu.edu.sa (L.A.A.); 2 Faculty of Medicine and Health, The University of Sydney, Sydney 2006, Australia; andrew.leaver@sydney.edu.au (A.L.); martin.mackey@sydney.edu.au (M.M.); 3 Department of Health and Rehabilitation Science, Prince Sattam Bin Abdulaziz University, Al-Kharj 16278, Saudi Arabia; a.alhowimel@psau.edu.sa (A.A.); maz.alotaibi@psau.edu.sa (M.A.); 4 Department of Physical Therapy, College of Applied Medical Sciences, Taif University, Taif 21944, Saudi Arabia; halzahrani@tu.edu.sa; 5 Physical Therapy Department, King Fahad Medical City, Riyadh 12231, Saudi Arabia

**Keywords:** pain behavior, low back pain, disability, psychosocial factors

## Abstract

**Background:** The Pain Behavioral Scale (PaBS) measures the presence and severity of pain behavior. We examine the longitudinal construct validity of the PaBS using convergent and known-groups approaches on a population of 23 participants with chronic lower back pain (LBP) undergoing routine physiotherapy care and pain neuroscience education. **Methods:** Participants who satisfied study inclusion and exclusion criteria were recruited from patients who attended two testing sessions at physiotherapy clinics in Saudi Arabia. Participant pain behavior was initially measured using the PaBS scale; participants performed standardized physical tests (e.g., repeated trunk flexion) and provided baseline demographic, clinical data, and self-reported measurements using the Modified Roland and Morris disability questionnaire (MODI), fear-avoidance questionnaire (FABQ), and pain catastrophizing scale (PCS). In subsequent visits, a physiotherapist provided usual care to participants, and weekly sessions were established for online pain-neuroscience education. During week six, participants repeated the same questionnaires and physical performance tests with the PaBS. Paired *t*-tests are used to compare changes in health characteristics from baseline responses to those in week six. Correlations between changes in PaBS from baseline to week six, with changes in outcome measures (i.e., disability, pain intensity, fear-avoidance beliefs, catastrophizing), were determined. To assess known-group validity, we also used a general linear model. **Results:** A total of 23 participants completed the PNE and follow-up data collection. The mean change from baseline in the PaBS score was statistically significant, as were changes in MODI, FABQ, and PCS. Almost 70% of participants improved their PaBS scores over the six-week period, with PaBS scores of almost 40% of them improving by three units or more. The change in PaBS score correlated significantly with changes in the PCS-rumination subscale, supporting a proposed approach to estimate convergent validity (r = 0.44, 95% CI = 0.04–0.72, *p* = 0.035). **Conclusions:** The mean change from baseline in the PaBS score is statistically significant, as are changes in MODI, FABQ, and PCS, supporting its convergent validity. According to our STarT Back groups, the medium to low-risk group had a lower PaBS score, and high-risk group had a higher PaBS score, indicating that PaBS use in clinical assessment may identify people according to pain-behavior severity, or those at increased risk of developing disability.

## 1. Introduction

Low back pain (LBP) is a multidimensional health problem that results in pain and disability [1]. Many psychosocial factors and pain-related beliefs, such as higher pain-related fear, psychological distress (e.g., anxiety or depression), and pain catastrophizing are risk factors for persistent or disabling LBP in older adults [2,3,4,5]. Longitudinal studies have revealed older persons with higher baseline fear-avoidance and depressive symptom scores are more likely to have disabling LBP at follow-up [6,7]. However, while psychosocial and cognitive aspects of LBP are well studied, the behavioral component related to chronic pain is not [8].

Observed pain-related behaviors are defined by the way symptoms are perceived and communicated to significant others [9]. Pain behaviors are common in people with chronic pain conditions [10,11,12,13,14,15,16,17,18]. According to the biopsychosocial model of pain, people with catastrophizing responses to pain may display more communicative pain behaviors during medical examination when a family supporter is present [19,20]. Numerous studies have reported a significant contribution of pain behavior to LBP-disability, as well as to other chronic conditions, such as knee osteoarthritis [11,12,21,22,23,24,25,26,27]. As such, assessing and targeting observable pain behaviors is an important part of a multidimensional pain-management plan [28].

The recently developed Pain Behavioral Scale (PaBS) is a clinician-based assessment that measures the presence and severity of observed pain behaviors [29]. While an exploratory study conducted among people with LBP demonstrated this scale to have excellent reliability and acceptable construct validity, further validation is warranted. No assessment of the predictive validity of the PaBS has been undertaken to measure longitudinal associations of pain-behaviors with individual, psychosocial factors, nor has its ability to detect change in a concept being measured (i.e., pain behavior) been reported. An ability to detect a change in pain behavior following a psychological intervention, such as pain-neuroscience education (PNE), is particularly important to provide a multidimensional understanding of LBP [30]. Strong evidence supports the use of PNE for musculoskeletal disorders to reduce pain severity, disability, pain catastrophizing, fear-avoidance, and pain-related behaviors [31].

We investigate the PaBS scale in a longitudinal study to appraise how well it can predict a patient’s clinical outcome. Our aims are to examine the longitudinal construct validity of the PaBS scale using convergent and known-groups approaches on an LBP population undergoing regular physiotherapy care and PNE. We hypothesize that changes in the PaBS will manifest moderate positive correlations with changes in disability-related to LBP, and that clinically important improvements in related LBP domains (including pain catastrophizing, fear-avoidance, pain intensity, and disability) will be associated with corresponding clinically important changes in pain behavior (as measured by PaBS).

## 2. Methods

### 2.1. Design and Setting

The longitudinal research design involved a sample of 23 adults with chronic LBP, attending one primary and one secondary care physiotherapy setting in Riyadh, Saudi Arabia, from February to September 2021. Ethics approval was granted by King Fahad Medical City Institutional Review Board (IRB 20-677E). This study is reported in accordance with the STROBE statement for the reporting of observational studies.

### 2.2. Participants

Individuals over 18 years of age, with LBP lasting more than 3 months, were recruited from consecutive patients who sought care for LBP and presented in two physiotherapy clinics. Participants were referred by physicians, pain clinic physicians, or had self-referred to the physiotherapy department of each clinic for physiotherapy. Each was provided with information about the study during their initial appointment, then screened using inclusion or exclusion criteria. All participants provided written informed consent prior to testing.

### 2.3. Inclusion and Exclusion Criteria

Study inclusion criteria included: age (18+) and having been diagnosed with chronic non-specific LBP for 3 or more months and having been screened at either of the clinics for inclusion suitability. Exclusion criteria included: having clinical features of serious pathology (e.g., malignancy, infection, inflammatory disorders or fracture, and spinal cord or cauda equina syndrome), specific pathologies causing LBP such as lumbar radiculopathy, pregnancy, an inability to complete written questionnaires in Arabic, lack of access to the internet to participate in online pain neurosciences education, and no mobile phone to receive reminder messages regarding the program’s online educational component.

### 2.4. Study Procedure

Participants were screened for eligibility by the research staff (i.e., examiners) using a standardized screening form [29]. Participants attended the clinic for two testing sessions: weeks 1 (baseline) and 6 (follow-up) for pain-behavior measurement using the PaBS [29]. The examiner rated the severity of select pain behavior in a series of standardized physical tests.

During week 1, participants provided baseline demographic and clinical data, self-reported measurements (including the level of study-specific psychosocial factors), pain intensity, and their level of LBP-related disability. A physiotherapist then assessed their risk of chronicity using the STarT Back Tool [32]. Participants performed a standardized sequence of physical performance tests, where their pain behaviors were rated using the PaBS by the treating physiotherapist (File S1) [29].

In subsequent visits during weeks 2 to 5, the physiotherapist provided usual care to participants. PNE were recorded in four videos by a certified specialist in pain neuroscience education (FA). PNE sessions (4 × 9–14 min videos) in Arabic were placed online for participant direct access. Weekly online PNE sessions were conducted by a research assistant, who contacted a participant up to twice (if necessary) weekly via text to provide a link to online sessions. Participants were asked to view the educational material at their convenience that week, and then they were asked to complete a related quiz.

During week 6, participants were given the same questionnaires they had completed in week 1 and performed the same physical performance tests with the PaBS to determine the presence and severity of pain behaviors. For completeness, we provide details of physical performance tests in File S1, but not their analysis.

### 2.5. Self-Report Measurements

Demographic characteristics (age, gender, marital status, education, smoking status, and work-related information) were collected on a standardized form [29].

Self-reported disability was assessed using the Modified Oswestry Disability Index (MODI) [33], and current pain intensity using the Numeric Pain Scale (NPS) [34]. The Arabic MODI comprises eight items related to physical function. Scores are calculated out of 100, with those > 21 indicating moderate disability [33,35]. Participants were asked to rate their current pain intensity by selecting a number between 0 (no pain at all) and 10 (the worst pain ever possible) that best corresponded to their pain level.

Pain-related fear was assessed using the Arabic fear-avoidance belief questionnaire (FABQ) [36], with 16 statements requiring a response; participants rated their agreement with each statement on a 7-point Likert scale (i.e., 0 = completely disagree, 7 = completely agree). The higher a score, the stronger a respondent’s fear-avoidance belief; the maximum score is 96. The FABQ includes two subscales: work (FABQ-w) with 7 questions and a maximum score of 42, and physical activity (FABQ-pa) with 4 questions and a maximum score of 24 [37].

Catastrophizing was evaluated using the Arabic pain catastrophizing scale (PCS) [38], which includes 13 items that assess a respondent’s thoughts and feelings towards their pain. The PCS consists of three subscales: rumination (4 items; questions 8–11), magnification (3 items; questions 6, 7, 13), and helplessness (6 items; questions 1–5, 12). Participants rated their thoughts and feelings regarding their pain for each PCS item on a 5-point Likert-type scale (0 = not at all, 1 = to a slight degree, 2 = to a moderate degree, 3 = to a great degree, 4 = all the time) [38,39]. A total PCS score was computed by summing all scores for all items, with higher scores indicating a higher tendency to catastrophize pain. Scores for the three PCS subscales were similarly obtained. A total PCS score ≥ 24 suggests a clinically relevant level of catastrophizing [40].

### 2.6. Pain-Related Behavior Measurement

The PaBS was used to record the presence and severity of pain behaviors during the performance of a standardized sequence of physical performance tests. Specific pain behaviors that were assessed, included sighing, breath-holding, grimacing, guarding, rubbing, and the occurrence of an antalgic gait. This 4-point scale ranges from “none” (no observed behavior) to “severe” (marked pain behavior). The total score of severity (0–15) was determined by summing the individual ratings of severity for pain behaviors observed for each test; a higher total score indicates greater severity of observed pain behaviors [29]. The physical performance tests included the following:

(1) Repeated trunk flexion: the time taken in seconds (s) for a participant to flex to the limit of their range of motion and return to an upright position 10 times, as fast as tolerable.
(2) Repeated sit to stand: the time taken (s) to rise to stand and return to sitting 5 times, as fast as possible.
(3) Timed up and go: the time taken (s) to rise from the seated position, stand and walk forward to a line 3 m away, turn, walk back to the chair, and sit down.
(4) Loaded reach: a participant stands next to a wall and holds a weight by their side not exceeding 5% of their body mass, and then reaches forward at shoulder height with the load. The maximum (loaded) reach distance (cm) is recorded.
(5) 50-foot (15.24 m) walk: the time taken (s) to walk 25 feet (7.62 m), turn around, and walk back to the starting position, as fast as possible.

### 2.7. Online Pain Neuroscience Educational Sessions

PNE sessions (4 × 9–14 min videos) were recorded in Arabic and placed online for participant access during weeks 2–5. Videos sought to reconceptualize pain, as well as to reduce fear, catastrophizing, and maladaptive behaviors associated with LBP disability by explaining the pain experience from a modern neuroscience perspective [41]. Video content included: in week 2, a discussion of pain neurophysiology was presented, contrasting the biomedical views of back pain with more holistic biopsychosocial views of the pain experience, as well as the definition of pain according to the 2020 International Association for the Study of Pain [42]; in week 3, biopsychosocial models of pain were presented, as well as psychosocial factors and beliefs contributing to the pain experience; in week 4, plasticity of the nervous system was presented; and, in week 5, the importance of behavioral changes to improve function was presented (https://youtu.be/ZkZhpSXyk5s (accessed on 14 July 2021)).

## 3. Data Analysis

Demographic, clinical, psychosocial, and other features of the health of participants were summarized using descriptive statistics with continuous variables reported as mean (μ), minimum and maximum standard deviation (SD), and categorical variables reported as frequency distributions (n, percent). Paired t-tests were used to compare changes in psychosocial and other health characteristics from baseline responses to those in week 6.

Longitudinal construct validity was examined using convergent and known-group approaches [43,44,45,46]. Convergent validity is an aspect of construct validity determined by correlating the measure of interest with other measures that we anticipate would produce similar results [44,47]. We evaluated convergent validity by examining correlations between changes in PaBS from baseline to week 6 with changes in outcome measures (i.e., disability, pain intensity, fear-avoidance beliefs, catastrophizing). Pearson rank correlation coefficients (r) are categorized as negligible (r < 0.1), small (0.1 ≤ r < 0.3), moderate (0.3 ≤ r < 0.5), or large (r ≥ 0.5) [48]. We hypothesized that changes in PaBS would manifest moderate positive correlations with changes in outcome measures (i.e., 0.3  ≤ r <  0.5) given that the clinical measure of interest (e.g., pain-behavior) increased the LBP-related disability.

Known-group validity can be examined by identifying differences in measures of interest between groups that are expected to score differently [44,47]. In a longitudinal context, known-group validity can be investigated by examining the mean change in a measure of interest in groups known to have experienced change in an underlying construct. We used the STarT Back Screening Tool to subgroup participants to explore possible differences in PaBS scores and to ascertain if a clinically important change in pain behavior scores between groups has occurred [44,47]. We categorized the STarT Back subgroups as “high-risk” (overall score > 3, psychosocial subscale score < 4), as well as “medium to low risk” (overall score 0–3, psychosocial subscale score ≤ 3), following Hill et al. [49] We identified two hypotheses a priori to determine known group validity.

High-risk group participants are more likely to have higher PaBS scores at baseline than medium to low-risk participants. As such, the STarT Back Screening Tool may discriminate between pain-behavior scores in a similar way to reference measures (e.g., disability, catastrophizing, fear) [50].

There will be a smaller between-group difference in the mean change from baseline PaBS score amongst high-risk-rated participants compared with medium- to low-risk-rated participants.

For hypothesis 1, known-group validity was assessed by a general linear model where categorical terms can be fitted, as well as to determine if mean baseline PaBS scores differed between STarT Back subgroups prior to and after adjustment for age and gender. A similar approach was taken for hypothesis 2 to determine if the mean change in PaBS scores differed between STarT Back subgroups before and after adjustment for age and gender.

Fitted models were:

$$Y_{i}=\beta_{0}+\beta_{1}STartBack\;subgroup$$

and

$$Y_{i}=\beta_{0}+\beta_{1}Age+\beta_{2}Gender+\beta_{3}\;STartBack\;subgroup$$

where: *Y_i_* is the baseline PaBS score for hypothesis 1, as well as the change from the baseline score for hypothesis 2. Age is fitted as a continuous variable, and gender and STarT Back subgroups are categorical variables [44].

Sensitivity analyses were undertaken for construct and known-group validity estimates. For construct validity, frequency tables were generated. Changes in PaBS, NPS, and MODI categories were classified by the size of any change from baseline values for those who had improved (i.e., score at week 6 was < week 1) by 1 or 2 units, 3 or 4 units or >4 units, those who had no change in score, and those whose scores worsened (i.e., score at week 6 was > week 1) by 1 or 2 units, or >2 units, respectively. For remaining instruments, improvement was categorized as a change of 1–10, 11–20, 21–30, and >30 units, no change, and worsening of 1–10 or 11–20 units.

For known-group validity, differences between baseline and week 6 STarT Back subgroup scores for each behavioral domain were investigated, similarly to the investigation for PaBS. That is, fitted models were:

$$Y_{i}=\beta_{0}+\beta_{1}STartBack\;subgroup$$

and

$$Y_{i}=\beta_{0}+\beta_{1}Age+\beta_{2}Gender+\beta_{3}STartBack\;subgroup$$

where: *Y* = the baseline score or change from baseline score, *i* = each behavioral pain domain (i.e., NPS, MODI, FABQ total score, FABQ-W, FABQ-PA, PCS total score, PCS rumination, PCS magnification, and PCS helplessness scores), and STarT Back subgroup = high risk, or medium to low risk.

As an exploratory study, we sought to recruit a sample size of 50 participants to detect a correlation between (r = 0.2–0.5) with a two-sided alpha = 0.05. This was not possible, and our sample population that met inclusion criteria was limited to 23 participants.

## 4. Results

All 23 enrolled participants completed the PNE and follow-up data collection. Demographic and clinical characteristics are reported in Table 1. On average, participants experienced a moderate level of pain (mean (SD) 4.3 (2.5)) and moderate level of disability (mean (SD) 37.1 (16.3)). Using the STarT Back tool, five participants were classified as low risk of disability, and nine each were categorized as medium and high risk. According to a previously identified cut-off point [39,51], participants had relatively low scores for fear-avoidance beliefs in both physical activity and work scales, as well as for pain catastrophizing.

A synopsis of changes in participant scores (difference in scores between weeks 1 and 6) in health characteristics are reported in Table 2. There was a small and statistically significant (*p* < 0.01) improvement in PaBS scores (average difference −1.9 (95% CI: −3.1 to −0.7) from baseline (average (SD) = 3.3 (2.75), Table 1) after completion of the six-week PNE program (Table 2). There was a significant (*p* < 0.01) improvement in disability (average difference of −13.8 (95% CI: −23.0 to −4.6)) and in fear-avoidance beliefs (−19.1 (−28.1, −10.1)) and pain catastrophizing (−8.0 (−12.0, −4.1)), both having *p* values < 0.001.

### 4.1. Longitudinal Construct Validity

Change in the PaBS score correlated significantly with changes in the PCS rumination subscale (r = 0.44, 95% CI = 0.04–0.72, *p* = 0.035) (Table 3, File S2). All remaining correlations with PaBS were small to negligible, and they were not statistically significant.

Results for sensitivity analysis are presented in File S3. Almost 70% of participants improved their PaBS scores over the six-week period, with the PaBS scores of almost 40% of them improving by three units or more. Although there was no significant change in the NRS score, the perception of pain of >60% of participants reduced over the 6 weeks; almost 50% of participants improved in the MODI category, >60% improved in their MODI score, with >26% improving their score by 30 points or more. Nearly 70% of participants improved their PCS rumination score, and >60% improved their FABQ physical-activity score.

### 4.2. Known-Groups Validity

General linear model results are presented in Table 4. Differences between mean baseline PaBS scores for each STarT Back subgroup were not significant, whether unadjusted or adjusted for age and gender, with no indication that any difference was clinically meaningful (differing by 1 unit).

## 5. Discussion

We sought to evaluate the longitudinal construct validity of the PaBS using convergent and known-group approaches on a LBP population, which was subject to usual physiotherapy care and PNE. We report the mean change in the PaBS score from baseline values to be statistically significant, as were changes in MODI, FABQ, and PCS, supporting their convergent validity. The PaBS score change also correlated significantly with changes in the pain catastrophizing rumination subscale.

A positive association exists between pain catastrophizing and pain behaviors [52,53,54,55]. This pattern of association is meaningful, because these two measures assess related underlying cognitive constructs [56]. For example, thoughts, such as ‘my pain is terrible’ or ‘pain will ruin my life’, can amplify symptoms [57], negatively affect problem solving and prevent positive behaviors [58,59], and assist with development of communicative pain behaviors [60]. Rumination, the repetitive and passive focusing on distress symptoms and their possible causes and consequences, such as pain behavior [58], is mainly triggered and resolved by pain itself [61]. A sense of danger and harm associated with rumination might explain the robust correlation that we report between pain catastrophizing and pain behaviors.

Because no comparable tool measures the severity of pain behavior [62], we explore the convergent validity of PaBS using tools that can measure related constructs, such as disability, fear avoidance, and pain catastrophizing. Although small, the statistically significant mean change from baseline in PaBS supports our proposed approach to estimate convergent validity. We also report changes from baseline scores in MODI, FABQ, and PCS to be statistically significant, indicating that the PaBS can measure behavioral change over time, even with small samples. Further, changes in the scores in these measures identified in this study can be explained within the biopsychosocial model of pain.

Communication of an individual’s pain as an observed pain behavior depends on the interpretation or meaning of pain (e.g., harmful vs normal). According to the fear-avoidance model of pain, when an activity is perceived as a risk, fear of future pain or recurrence of pain may develop [9,62]. Fear may result in behavioral actions (e.g., avoidance), and it can lead to catastrophic thinking that might impact other pain-related behaviors (e.g., grimacing, sighing) [63]. As such, assessing pain behaviors is an important part of multidimensional LBP management.

Our data do not show the PaBS to be capable of differentiating people of high and medium–low risk, as assessed using the STarT Back Screening Tool. However, while differences between mean baseline PaBS scores for each STarT Back subgroup were not significant, medium–low-risk groups had lower PaBS scores than the high-risk group. As such, pain behaviors may aid with the identification of individuals at increased risk of developing LBP-related disability [17]. Our results suggest that PaBS use in clinical assessment may be able to identify people according to the severity of pain-behavior, or those at increased risk of developing disability [17], and it may facilitate the targeting of underlying mechanisms [22,64,65,66].

In summary, we provide evidence to support some aspects of the longitudinal construct validity of the PaBS scale, which was earlier found to have an acceptable cross-sectional construct validity [29]. Unfortunately, period constraints (COVID-19 restrictions, lockdowns, and fewer patients attending clinics) limited the number of participants in our study and affected our sample size and statistical confidence intervals. Associated with small sample size are limitations in the extent to which results can be generalized to broader populations, low statistical power, and the increased likelihood of a type II error. For instance, low sample size in the STarT Back analysis (Table 4) may have led to some differences between these two groups going undetected (type II error). Obviously, a more-informed critique of the effectiveness of the PaBS scale requires a larger sample size (or multicenter study) [67,68], but circumstances beyond our control precluded this. Given the planning, investment in resources and personnel, and extenuating circumstances, we elected to proceed with this study because this research formed an integral part of a larger academic program, informed it (being exploratory), and, ultimately, the data that it provided may prove to be of historical importance in a larger post-COVID-19-pandemic survey. Further analysis with more participants and a more-representative sample of the LBP population would enable the correlation that we report between pain behavior with catastrophizing to be independently critiqued.

## 6. Conclusions

Support for the longitudinal validity of the PaBS scale is reported. In addition to the mean change in the PaBS score from baseline values being statistically significant so too are changes in disability, fear avoidance beliefs and pain catastrophizing. The PaBS scale can be used as a screening tool in clinical practice to assist with the assessment and monitoring of pain behaviors in people with LBP. 

## Figures and Tables

**Table 1:** Participant demographic and clinical characteristics.

		STarT Back Subgroups		
	Total (n = 23)	Low (n = 5)	Medium (n = 9)	High (n = 9)
Age mean (SD)	39.4 (11.74)	43.2 (13.77)	38.3 (13.01)	38.3 (10.19)
Minimum–maximum	23–58	28–58	23–56	26–52
Sex n (%)				
Female	16 (69.6)	5 (100)	6 (66.7)	5 (55.6)
Marital status n (%)				
Married	16 (69.6)	3 (60.0)	8 (88.9)	5 (55.6)
Education level n (%)				
Elementary/high School	4 (17.4)	2 (40.0)	2 (22.2)	0
Diploma Degree	4 (17.4)	0	1 (11.1)	3 (33.3)
Academic Degree	15 (65.2)	3 (60)	6 (66.7)	6 (66.7)
Smoker n (%)				
No	19 (82.6)	5 (100)	7 (77.8)	7 (77.8)
Employment Status ^1^ n (%)				
Unemployed	9 (39)	2 (40.0)	3 (33.3)	4 (44.4)
Employed	14 (61)	3 (60.0)	6 (66.7)	5 (55.6)
Baseline Disability (MODI-)				
mean (SD)	37.1 (16.30)	19.5 (6.47)	43.1 (12.73)	40.8 (17.28)
Minimum–maximum	10–67.5	10–27.5	30–67.5	17.5–60
Baseline Disability (MODI- categories) n (%)				
Minimal	6 (26.1)	3 (60.0)	0	3 (33.3)
Moderate	9 (39.1)	2 (40.00)	6 (66.7)	1 (11.1)
Severe and extremely severe	8 (34.8)	0	1 (11.1)	5 (55.6)
Baseline Pain intensity (NRS) (0–10)				
mean (SD)	4.3 (2.51)	3.2 (2.17)	5.2 (1.99)	3.9 (3.02)
Minimum–maximum	0–8	0–6	2–8	0–8
Baseline Pain behavior scale (PaBS) (0–15)				
mean (SD)	3.3 (2.75)	2.6 (2.30)	3.6 (1.67)	3.4 (3.88)
Minimum-maximum	0–10	1–6	1–7	0–10
Baseline Fear avoidance belief (FABQ-physical activity) (0–26) ^2^				
mean (SD)	12.2 (6.78)	9.2 (5.81)	14.4 (6.35)	11.7 (7.60)
Minimum–maximum	0–23	4–18	1–23	0–20
Baseline Fear avoidance belief (FABQ-work) (0–42) ^2^				
mean (SD)	22.2 (8.58)	20.0 (9.72)	21.3 (8.51)	24.2 (8.61)
Minimum–maximum	3–34	11–34	3–33	6–33
Baseline Pain Catastrophizing Scale (PCS-total) (0–52) ^2^				
mean (SD)	17.9 (11.23)	7.4 (7.02)	22.1 (11.43)	19.6 (9.99)
Minimum-maximum	0–39	0–19	4–39	4–36
PCS-rumination subscale (0–16)				
mean (SD)	8.2 (4.24)	3.8 (2.86)	9.2 (4.06)	9.6 (3.68)
Minimum–maximum	0–15	0–8	2–15	2–15
PCS-magnification subscale (0–12)				
mean (SD)	2.8 (3.22)	1.8 (2.95)	3.4 (3.47)	2.7 (3.32)
Minimum–maximum	0–9	0–7	0–9	0–9
PCS-helplessness subscale (0–24)				
mean (SD)	7.0 (5.05)	1.8 (1.48)	9.4 (4.85)	7.3 (4.66)
Minimum–maximum	0–19	0–4	2–19	1–16
STarT Back Screen n (%)				
Low risk	5 (21.7)			
Medium risk	9 (39.1)			
High risk	9 (39.1)			

^1^ Unemployed includes those not working, students, and housewives. Employed includes teachers, engineers, and those in business administration. ^2^ A score for the FABQ-physical activity (>14) and a score for the FABQ-work (>29) are classified high or elevated baseline scores. A cutoff of more than 30 points in PCS is classified high or can be associated with clinical relevance.

**Table 2:** Synopsis of changes in participant scores for health characteristics between weeks one and six.

Instrument	Mean (SD)	95% CI
Pain intensity (NRS)	−1.2 (3.45)	−2.7, 0.3
Pain behavior scale (PaBS)	−1.9 (2.80) ^1^ **	−3.1, −0.7
Disability (MODI)	−13.8 (21.31) **	−23.0, −4.6
Fear avoidance belief		
FABQ—total score	−19.1 (20.85) ***	−28.1, −10.1
FABQ—physical activity	−4.4 (8.47) *	−8.1, −0.8
FABQ—work	−9.3 (11.64) ***	−14.3, −4.3
Pain Catastrophizing Scale		
PCS—total	−8.0 (9.2) ***	−12.0, −4.1
PCS—rumination	−2.7 (0.83) **	−4.5, −1.0
PCS—magnification	−1.5 (2.81) *	−2.7, −0.3
PCS—helplessness	−3.8 (4.57) ***	−5.8, −1.9

^1^ *p* value is from the paired *t*-test comparison of weeks 6 and 1. * *p* < 0.05; ** *p* < 0.01; *** *p* < 0.001.

**Table 3:** Correlations between change from baseline PaBS scores and those of other measures for longitudinal construct validity assessment.

Instrument	PaBS r (95% CI)
NRS	−0.11 (−0.50, 0.31)
MODI	0.03 (−0.39, 0.44)
Total FABQ	0.02 (−0.39, 0.43)
FABQ—work	−0.02 (−0.43, 0.39)
FABQ—physical activity	0.26 (−0.17, 0.60)
Total PCS	0.17 (−0.26, 0.55)
PCS—rumination	0.44 * (0.04, 0.72)
PCS—magnification	−0.27 (−0.61, 0.16)
PCS—helplessness	0.13 (−0.30, 0.51)

*p*-value tests the null hypothesis that the correlation with change from baseline PaBS score = 0. * *p* < 0.05.

**Table 4:** Difference between weeks one and six in behavioral pain scores and STarT Back subgroups for known-group validity assessment.

	STarT Back			STarT Back Adjusted for Age and Gender		
Characteristic	Low/Medium Risk Mean (95% CI)	High Risk Mean (95% CI)	*p* Value	Low/Medium Risk Mean (95% CI)	High Risk Mean (95% CI)	*p* Value
PaBS						
Baseline	3.2 (1.6, 4.8)	3.4 (1.5, 5.4)	0.85	4.1 (2.5, 5.7)	3.7 (1.9, 5.5)	0.73
Change from baseline	−1.6 (−3.2, 0)	−2.3 (−4.3, −0.4)	0.53	−2 (−3.8, −0.2)	−2.4 (−4.4, −0.4)	0.77
Current pain						
Baseline	4.5 (3.1, 5.9)	3.9 (2.1, 5.7)	0.58	5 (3.5, 6.6)	4.1 (2.4, 5.8)	0.37
Change from baseline	−0.6 (−2.5, 1.3)	−2.2 (−4.6, 0.2)	0.27	−1.5 (−3.5, 0.4)	−2.5 (−4.7, −0.3)	0.49
MODI score						
Baseline	34.6 (25.5, 43.7)	40.8 (29.5, 52.2)	0.38	37.2 (26.8, 47.6)	41.2 (29.6, 52.9)	0.58
Change from baseline	−7.3 (−18.5, 3.8)	−23.9 (−37.8, −10)	0.06	−13 (−23.4, −2.6)	−24.3 (−35.9, −12.7)	0.13
FABQ—total score						
Baseline	41.7 (32.8, 50.7)	45.7 (34.5, 56.8)	0.57	42.4 (31.9, 52.9)	45.6 (33.9, 57.4)	0.66
Change from baseline	−10.3 (−20.3, −0.3)	−32.8 (−45.2, −20.3)	0.01	−13.1 (−24.3, −1.9)	−33 (−45.6, −20.5)	0.02
FABQ—work score						
Baseline	20.9 (16.1, 25.6)	24.2 (18.3, 30.2)	0.37	21.3 (15.8, 26.9)	24.2 (17.9, 30.4)	0.47
Change from baseline	−4.3 (−9.8, 1.2)	−17.1 (−24, −10.2)	0.01	−6 (−12.3, 0.2)	−17.4 (−24.3, −10.4)	0.02
FABQ—PA score						
Baseline	12.6 (8.7, 16.4)	11.7 (6.9, 16.5)	0.76	13.2 (8.7, 17.7)	11.7 (6.7, 16.7)	0.64
Change from baseline	−2.5 (−7.1, 2.1)	−7.6 (−13.3, −1.8)	0.16	−3.8 (−9, 1.3)	−7.7 (−13.4, −1.9)	0.30
PCS total score						
Baseline	16.9 (10.5, 23.2)	19.6 (11.6, 27.5)	0.58	19.9 (13.2, 26.6)	20.1 (12.6, 27.6)	0.97
Change from baseline	−5.1 (−9.9, −0.3)	−12.6 (−18.5, −6.6)	0.05	−8.7 (−12.9, −4.6)	−13.2 (−17.8, −8.6)	0.14
PCS rumination score						
Baseline	7.3 (5, 9.6)	9.6 (6.7, 12.5)	0.22	8.3 (5.8, 10.8)	9.7 (6.9, 12.5)	0.43
Change from baseline	−1 (−2.9, 0.9)	−5.4 (−7.8, −3.1)	0.01	−2.3 (−4, −0.6)	−5.6 (−7.5, −3.8)	0.01
PCS magnification score						
Baseline	2.9 (1, 4.7)	2.7 (0.4, 4.9)	0.89	3.6 (1.5, 5.6)	2.8 (0.5, 5)	0.58
Change from baseline	−1.6 (−3.2, 0)	−1.3 (−3.3, 0.7)	0.84	−2.3 (−4, −0.6)	−1.4 (−3.3, 0.5)	0.49
PCS helplessness score						
Baseline	6.7 (3.8, 9.6)	7.3 (3.8, 10.9)	0.78	8 (4.9, 11.1)	7.6 (4.1, 11)	0.83
Change from baseline	−2.6 (−5, −0.1)	−5.8 (−8.8, −2.7)	0.10	−4.2 (−6.5, −1.9)	−6.1 (−8.7, −3.6)	0.25

## Data Availability

Due to privacy and ethical concerns, our data cannot be made available online.

## Supplementary Materials

The following supporting information can be downloaded at: https://www.mdpi.com/article/10.3390/healthcare11121743/s1, File S1: Table S1. Components of physical performance tests and PaBS descriptive data; Table S2. Components of physical performance tests and PaBS descriptive data (follow-up); Table S3: Frequency of the pain related behaviors during physical performance tests (Baseline); Table S4: Frequency of the pain related behaviors during physical performance tests (follow-up); Table S5: Differences in physical performance tests and PaBS descriptive data; File S2: Correlations between change from baseline PaBS scores and those of other measures for longitudinal construct validity assessment; File S3: Frequency and percentage of participants showing improvement, no change, or worsening behavior for each instrument.
