# Supplementary material for: An Assessment of the Longitudinal Construct Validity of the Pain Behavioral Scale (PaBS) in a Saudi Population with Chronic Low Back Pain: A Preliminary Study
Source: Healthcare (Basel). 2023 Jun 14;11(12):1743. doi: 10.3390/healthcare11121743 (PMC10297879; doi:10.3390/healthcare11121743)
Supplement: Supplementary file 1 [file healthcare-11-01743-s001.zip › File S1.pdf]

File S1

Table S1. Components of physical performance tests and PaBS descriptive data

	Minimum	Maximum	Mean	Std. Deviation
Trunk flexion (sec)	15	52	29.80	9.98
Sit to stand (sec)	7	24	16.34	4.35
Timed up and go (sec)	6	16	9.16	2.64
Loaded reach (cm)	21	39	29.95	5.49
50-foot walk (s)*	9	37	13.86	5.89

(Baseline)

Table S2. Components of physical performance tests and PaBS descriptive data (follow-up)

	Minimum	Maximum	Mean	Std. Deviation
Trunk flexion (sec)	10	38.28	25.28	7.13
Sit to stand (sec)	5	27	14.80	4.38
Timed up and go (sec)	6	15	8.93	2.18
Loaded reach (cm)	18	47	29.30	6.23
50-foot walk (sec)	8	34	12.67	5.30

Table S3: Frequency of the pain related behaviors during physical performance tests (Baseline)

	Sighing (yes)	Breath-hold (yes)	Grimacing (yes)	Grimacing (yes)	Rubbing (yes)	Antalgic gait (yes)
Trunk flexion, n (%)	7 (30.4)	9 (39.1)	4 (17.4)	0	0	NA*
Sit to stand, n (%)	4 (17.4)	3 (13)	2 (8.7)	0	0	NA*
Timed up and go, n (%)	1 (4.3)	1 (4.3)	1 (4.3)	0	0	0
Loaded reach, n (%)	3 (13)	3 (13)	4 (17.4)	0	0	NA*
50-foot walk, n (%)	1 (4.3)	3 (13)	2 (8.7)	1 (4.3)	1 (4.3)	0

*NA: not applicable

Table S4: Frequency of the pain related behaviors during physical performance tests (follow-up)

	Sighing (yes)	Breath-hold (yes)	Grimacing (yes)	Grimacing (yes)	Rubbing (yes)	Antalgic gait (yes)
Trunk flexion, n (%)	7 (30.4)	4 (17.4)	0	0	0	NA*
Sit to stand, n (%)	1 (4.3)	3 (13)	1 (4.3)	0	0	NA*
Timed up and go, n (%)	0	0	0	0	0	2 (8.7)
Loaded reach, n (%)	0	0	3 (13)	0	0	NA*
50-foot walk, n (%)	0	0	1 (4.3)	0	0	1 (4.3)

*NA: not applicable

Table S5: Differences in physical performance tests and PaBS descriptive data

	Baseline Mean (SD)	Follow-up Mean (SD)	Mean difference (95% CI)	*t*	*p* value
Trunk flexion (sec)	29.80 (9.98)	25.28 (7.13)	4.74 (1.45–8.04)	2.98	0.01
Sit to stand (sec)	16.34 (4.35)	14.80 (4.38)	1.53 (0.04–3.03)	2.13	0.04
Timed up and go (sec)	9.16 (2.64)	8.93 (2.18)	0.68 (−0.16–1.53)	1.66	0.11
Loaded reach (cm)	29.95 (5.49)	29.30 (6.23)	0.65 (−2.01–3.31)	0.50	0.61
50-foot walk (sec)	13.86 (5.89)	12.67 (5.30)	1.19 (−1.61–4)	0.88	0.38
