# Supplementary material for: An Assessment of the Longitudinal Construct Validity of the Pain Behavioral Scale (PaBS) in a Saudi Population with Chronic Low Back Pain: A Preliminary Study
Source: Healthcare (Basel). 2023 Jun 14;11(12):1743. doi: 10.3390/healthcare11121743 (PMC10297879; doi:10.3390/healthcare11121743)
Supplement: Supplementary file 1 [file healthcare-11-01743-s001.zip › Files S2 and S3.pdf]

File S2: Correlations between change from baseline PaBS scores and those of other measures for longitudinal construct validity assessment

				FABQ			PCS		
				total	W	PA	total	rumination	magnif.
Instrument	PaBS	NPS	MODI						
Change in measure	r<br>(95% CI)	r<br>(95% CI)	r<br>(95% CI)	r<br>(95% CI)	r<br>(95% CI)	r<br>(95% CI)	r<br>(95% CI)	r<br>(95% CI)	r<br>(95% CI)
NPS	−0.11								
	(−0.50, 0.31)								
MODI	0.03	0.45*							
	(−0.39, 0.44)	(0.05, 0.73)							
Total FABQ	0.02	0.47*	0.73***						
	(−0.39, 0.43)	(0.08, 0.74)	(0.46, 0.88						
FABQ W	−0.02	0.39	0.72***	0.86***					
	(−0.43, 0.39)	(−0.02, 0.69)	(0.44, 0.87)	(0.69, 0.94)					
FABQ PA	0.26	0.41	0.55**	0.73***	0.35				
	−0.17, 0.60)	(−0.01, 0.70)	(0.17, 0.78)	(0.46, 0.88)	(−0.07, 0.67)				
Total PCS	0.17	0.55**	0.53**	0.42*	0.48*	0.22			
	(−0.26, 0.55)	(0.18, 0.79)	(0.15, 0.77)	(0.01, 0.71)	(0.09, 0.75)	(−0.21, 0.58)			
PCS rumination	0.44*	0.43*	0.50*	0.49*	0.60*	0.28	0.83***		
	(0.04, 0.72)	(0.03, 0.72)	(0.11, 0.76)	(0.10, 0.75)	(0.25, 0.81)	(−0.15, 0.62)	(0.63, 0.92)		
PCS magnification	−0.27	0.31	0.14	0.02	0.11	−0.16	0.66***	0.33	
	(−0.61, 0.16)	(−0.11, 0.64)	(−0.29, 0.52)	(−0.40, 0.43)	(−0.32, 0.50)	(−0.53, 0.27)	(0.34, 0.84)	(−0.10, 0.65)	
PCS helplessness	0.13	0.54**	0.55**	0.41	0.38	0.31	0.89***	0.59**	0.43*
	−0.30, 0.51)	(0.17, 0.78)	(0.18, 0.79)	(−0.0003, 0.70)	(−0.04, 0.69)	(−0.12, 0.64)	(0.75, 0.95)	(0.24, 0.81)	(0.02, 0.72)

File S3: Frequency and percentage of participants showing improvement, no change, or worsening behavior for each instrument

Instrument	Change	Amount of change	Frequency (n)	Percent (%)
PaBS Change Score	Improvement	> 4 units	2	8.7
		3 to 4 units	7	30.4
		1 to 2 units	7	30.4
	No change	0 units	2	8.7
	Worsening	1 to 2 units	5	21.7
NPS Change Score	Improvement	> 4 units	5	21.7
		3 to 4 units	2	8.7
		1 to 2 units	7	30.4
	No change	0 units	4	17.4
	Worsening	1 to 2 units	2	8.7
		> 2 units	3	13.0
MODI Change category	Improvement	1 to 2 units	11	47.8
	No change	0 units	7	30.4
	Worsening	1 to 2 units	5	21.7
MODI Change Score	Improvement	> 30 units	6	26.1
		21 to 30 units	3	13.0
		11 to 20 units	2	8.7
		1 to 10 units	3	13.0
	No change	No change	1	4.4
	Worsening	1 to 10	6	26.1
		11 to 20	2	8.7
FABQ Total Change	Improvement	> 30 units	7	30.4
		21 to 30 units	2	8.7
		11 to 20 units	5	21.7
		1 to 10 units	4	17.4
	Worsening	1 to 10	3	13.0
		11 to 20	2	8.7
FABQ-W Change	Improvement	> 30 units	1	4.4
		21 to 30 units	5	21.7
		11 to 20 units	4	17.4
		1 to 10 units	6	26.1
	No change	0 units	1	4.4
	Worsening	1 to 10	6	26.1
FABQ-PA Change	Improvement	11 to 20 units	7	30.4
		1 to 10 units	7	30.4
	No change	0 units	1	4.4
	Worsening	1 to 10	8	34.8
PCS Total Change	Improvement	21 to 30 units	3	13.0
		11 to 20 units	4	17.4
		1 to 10 units	12	52.2
	Worsening	1 to 10	4	17.4
PCS Rumination Change	Improvement	1 to 10 units	16	69.6
	No change	0 units	2	8.7
	Worsening	1 to 10	5	21.7
PCS Magnification Change	Improvement	1 to 10 units	11	47.8
	No change	0 units	7	30.4
	Worsening	1 to 10	5	21.7

Instrument	Change	Amount of change	Frequency (n)	Percent (%)
PCA Helplessness Change	Improvement	11 to 20 units	2	8.7
		1 to 10 units	16	69.6
	No change	0 units	3	13.0
	Worsening	1 to 10	2	8.7
